# Advancements in Pharmacological Interventions and Novel Therapeutic Approaches for Amyotrophic Lateral Sclerosis

**DOI:** 10.3390/biomedicines12102200

**Published:** 2024-09-27

**Authors:** María Montiel-Troya, Himan Mohamed-Mohamed, Teresa Pardo-Moreno, Ana González-Díaz, Azahara Ruger-Navarrete, Mario de la Mata Fernández, María Isabel Tovar-Gálvez, Juan José Ramos-Rodríguez, Victoria García-Morales

**Affiliations:** 1 Faculty of Health Sciences Ceuta, University of Granada, 51001 Ceuta, Spain; mariamontiel@ugr.es (M.M.-T.); tpardo@ugr.es (T.P.-M.); agonzalezd@ugr.es (A.G.-D.); azahara.ruger@ugr.es (A.R.-N.); 2 Department of Physiology, Faculty of Health Sciences Ceuta, University of Granada, 51001 Ceuta, Spain; imanceuta@gmail.com (H.M.-M.); mrdelamata@ugr.es (M.d.l.M.F.); juanjoseramos@ugr.es (J.J.R.-R.); 3 Nursing Department, Faculty of Health Sciences, University of Granada, Avda. Ilustración 69, 18071 Granada, Spain; 4 Department of Biomedicine, Biotechnology and Public Health, Physiology Area, Faculty of Medicine, University of Cádiz, Pl. Falla, 9, 11003 Cádiz, Spain; victoria.garcia@gm.uca.es

**Keywords:** ALS, amyotrophic lateral sclerosis, neurodegenerative disorder, motor neuron dysfunction, multidisciplinary treatment, pharmacological interventions

## Abstract

(1) Amyotrophic Lateral Sclerosis (ALS) is a neurodegenerative disease in which the patient suffers from an affection of both upper and lower motor neurons at the spinal and brainstem level, causing a progressive paralysis that leads to the patient’s demise. Gender is also considered a predisposing risk factor for developing the disease. A brief review of the pathophysiological mechanisms of the disease is also described in this work. Despite the fact that a cure for ALS is currently unknown, there exists a variety of pharmacological and non-pharmacological therapies that can help reduce the progression of the disease over a certain period of time and alleviate symptoms. (2) We aim to analyze these pharmacological and non-pharmacological therapies through a systematic review. A comprehensive, multidisciplinary approach to treatment is necessary. (3) Drugs such as riluzole, edaravone, and sodium phenylbutyrate, among others, have been investigated. Additionally, it is important to stay updated on research on new drugs, such as masitinib, from which very good results have been obtained. (4) Therapies aimed at psychological support, speech and language, and physical therapy for the patient are also available, which increase the quality of life of the patients.

## 1. Introduction

Amyotrophic Lateral Sclerosis (ALS), also known as motor neuron (MN) disease, is a heterogeneous, less prevalent, and fatal neurodegenerative disorder with an unknown etiology. It primarily affects the motor system, leading to a progressive loss of upper MNs in the cortex and lower MNs in the spinal cord and medulla oblongata. This causes a complex clinical picture in patients, resulting in difficulties in performing activities of daily living [1,2]. There are four variants of clinical presentation depending on which structures are most affected: Progressive Bulbar Palsy (PBP), Progressive Muscular Atrophy (PMA), Primary Lateral Sclerosis (PLS), and classic ALS, with the latter being the most common [3].

To understand the pathophysiology of ALS, it is vital to recognize the role of MNs since their degeneration leads to the symptoms of the disease. ALS may originate from the initial degeneration of either upper or lower MNs, affecting both pathways of motor control of the skeletal musculature. Therefore, the origin of the disease could be bulbar (when lower MNs are affected first) or non-bulbar (when upper MNs are affected first) [4]. There are two forms of ALS: familial and sporadic. Familial ALS is hereditary, accounting for 5–10% of cases, where a specific gene is responsible, while sporadic ALS, which does not have any linked genetic factors, accounts for 90–95% of cases [4,5]. In familial ALS, genetic mutations have been found on chromosome 21 in the gene encoding the Cu/Zn-dependent superoxide dismutase (SOD1) [3]. Additionally, mutations in TDP-43 and C9ORF72 have been identified. The cause of the disease is identifiable in familial ALS due to specific gene mutations, such as SOD1. The toxicity of this gene is not due to the loss of its function (catalyzing the conversion of superoxide anion O_2_^−^ to oxygen and hydrogen peroxide) but to the formation of mutagenic complexes that induce the death of upper MNs due to their high molecular weight.

Sporadic ALS is not caused by genetic mutations. Its origin is unknown, and there are numerous theories and hypotheses attempting to explain the cause of neuronal degeneration in affected patients. These include glutamate excitotoxicity and oxidative stress caused by an imbalance between the generation and elimination of free radicals. Due to the nervous system’s vulnerability to oxidative damage, this imbalance leads to neuronal dysfunction and neurodegeneration. Additionally, alterations in axonal transport, excitotoxicity caused by astrocyte malfunction, mitochondrial abnormalities in MNs, and glial cell level alterations have been described. Numerous studies have indicated differences in the prevalence of ALS among Hispanic populations (European descendants), African Americans, and Native Americans. This suggests a heterogeneous distribution of incidence due to the presence of subcontinents (territories with similar properties within a continent) and genetics [3]. From the onset of the disease, half of the patients die within 3 years, 80% within 5 years, and 95% within 10 years. Populations with more homogeneity, such as those of European origin (Europe, North America, and New Zealand), have an incidence of 1.81/100,000 inhabitants, which is 50 to 100 times higher than in other parts of the world [3,6].

In contrast, populations with less homogeneity (South and East Asia and South Korea) have an incidence of 0.6/100,000 inhabitants. The increase in ALS cases in these regions is attributed to environmental factors, specifically a non-protein neurotoxic amino acid called beta-methylamino-L-alanine (L-BMAA) [6]. This amino acid is produced by cyanobacteria and diatoms in terrestrial and aquatic ecosystems. When bioaccumulated through food webs, people ingest food, drink water, or inhale air from these environments, leading to contamination. This amino acid is highly excitotoxic and interacts with glutamate receptors, mimicking its action.

### 1.1. Pathophysiological Mechanisms in ALS

**Figure 1:**
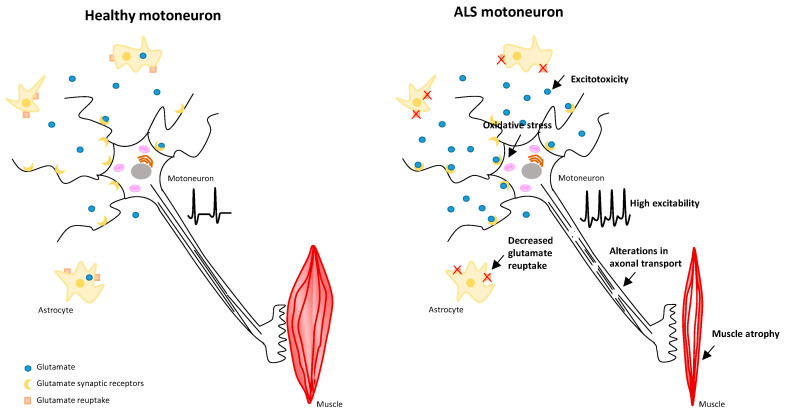
Schematic representation of the pathophysiological mechanisms underlying ALS.

Although the primary cause of ALS has not been elucidated, different hypotheses have been proposed to explain the mechanisms causing such damage in these patients. Various etiological investigations include both genetic and exogenous factors, which are not mutually exclusive. The mechanisms discussed in this review are represented in Figure 1. The most relevant findings include:

Glutamate excitotoxicity: Glutamate is the main excitatory neurotransmitter in the CNS. There are several types of glutamatergic receptors in MNs, such as AMPA (α-amino-3-hydroxy-5-methyl-4-isoxazolpropionic acid), NMDA (N-methyl-D-aspartate), and kainate receptors. The binding of glutamate to these receptors induces a depolarizing response in neurons, increasing their excitability due to the intracellular rise in calcium ions [7]. A sustained increase in neuronal excitability can lead to hyperexcitability, resulting in degeneration or death. Glutamate is produced as an intermediate metabolite in the Krebs cycle, specifically alpha-ketoglutarate. Elevated physiological levels or heightened sensitivity of MNs to glutamate cause excessive activation of specific receptors, including those mentioned above (located in the membranes of neurons and astrocytes around the neuronal synapse) [3,6]. Furthermore, in the cerebrospinal fluid of patients with ALS, glutamate levels are elevated due to impaired reuptake and loss of astrocyte transporters (EAAT2 has a greater affinity for glutamate, though EAAT1 is also present).

Oxidative stress: Oxidative stress occurs when there is an imbalance between the generation and elimination of free radicals or an impaired ability to repair or eliminate damage in MNs. Oxidative stress is involved in a wide variety of mechanisms, leading to the oxidation of nucleic acids, lipids, and proteins. These pathogenic mechanisms include the formation of reactive oxygen species (ROS) and reactive nitrogen species (such as nitric oxide, NO), mitochondrial dysfunction inducing alterations in the blood–brain barrier, deficits in antioxidant defenses, and the production of anti-inflammatory responses. This induces neuronal damage and promotes MN death [8]. Regarding the etiology, oxidative stress may be due to excessive ROS production, which may underlie mitochondrial dysfunction [9]. Overproduction of ROS may cause lipid peroxidation, protein oxidation, or DNA mutations. Mitochondrial dysfunction found in ALS includes deficiencies in ATP production [10], alterations in mitochondrial dynamics and recycling, promoting the accumulation of damaged mitochondria, and an increase in mitochondrial membrane permeability [11].

Alterations in axonal transport and mitochondrial dynamics: Neuronal deficiencies originating at the axonal level are considered early pathologies of this disease. According to Guo et al. [12], mutations in human genes are closely related to axonal transport and neurodegeneration. Various proteins are transported through axons, involving microtubules and associated motor proteins like dyneins and kinesins. Dynein is involved in retrograde transport, which begins in the axons toward the microtubules, while kinesins are involved in anterograde transport, starting from the cell body to the axon, opposite to retrograde transport [12,13,14]. ATP, synthesized by mitochondria, is crucial for metabolizing energy used by neurons and the axon. Alterations in mitochondria result in poor generation and regeneration of axonal damage [15,16]. Mitochondria are transported through the axon to meet the neuron’s energy demands. The biological energy of mitochondria and axonal transport, along with the coactivator PGC1α (peroxisome proliferator-activated receptor gamma coactivator 1-alpha), is essential for this process. Excessive expression of this coactivator would not favor the metabolism of mitochondria and the regeneration of axonal damage, highlighting the importance of balance [17].

Microglia and astrocytes: Neuroinflammation is a pathological mechanism in patients with ALS. Numerous authors suggest that neuroinflammation could be due to excessive activation of microglial cells and astrocytes [18]. Among their functions and properties, both have neuroprotection and neurotoxicity. These cells undergo functional alterations during the disease development. According to Brandebura et al. [18], astrocytes have the ability to switch from a non-reactive to a reactive form, called astrogliosis, in the face of a pathological stimulus from the environment by increasing glutamate in the extracellular space [3,19]. Microglial cells can be activated in a toxic environment because their function is to control the environment. When this happens, their shape and size vary, in addition to which they can engulf the toxic substance, dead cells, and waste found in the middle and eliminate it. It is possible that these cells lose their ability to phagocytize toxic products and, consequently, glial cells become reactive. By becoming reactive, they attract more astrocytes leading to constant inflammation [3,17]. This malfunction in astrocytes and microglia disrupts the physiological environment of MN somas, altering their function and homeostasis, and making them more susceptible to death by excitotoxicity [20].

### 1.2. Risk Factors in ALS

ALS presents a wide variety of factors that can be described and understood in terms of risk agents. Most patients with this disease are between 40 and 70 years of age. Despite this, several studies in patients with ALS have found relationships with the following factors. Genetic factors: Research has demonstrated that over 30 genes are associated with this disease; however, these genes do not directly initiate its onset. Mutations in the SOD1 gene (superoxide dismutase 1 enzyme) are related to familial ALS, involving only 20% of these cases. In addition, 10% of cases are related to the TDP-43 gene, and the FUS/BPD gene is associated with 5% of cases. In contrast, an abnormal expression of the C9ORF72 gene is significant for this disease.

Toxic habits: The consumption of tobacco has a negative effect on motor neurons, inducing neurotoxicity and acting directly on them. It also triggers oxidative stress, leading to the development of inflammatory processes in the body. A large number of ALS patients confirmed that they had smoked tobacco for at least three years at some point in their lives [3].

Environmental factors: Pollution from vehicle traffic, including cars, buses, and motorcycles, and exposure to particles in constant suspension in cities (carbon monoxide and nitrous oxides) increase the risk of ALS. Another environmental factor is exposure to heavy metals released naturally by industries or car engines. These metals contaminate aquifer surfaces, and when ingested or inhaled, they reach human organs. Heavy metals (Pb, Cd, Al, and Mg) are toxic and found in the blood. Additionally, exposure to pesticides (herbicides, insecticides, or fungicides) by farmers for crop development and care is also neurotoxic.

Diabetes mellitus: According to a study by Wannarong et al. [21] diabetes mellitus is a protective factor in patients with ALS. The exact pathophysiological mechanisms are unknown, but it is believed to be related to neuronal hypermetabolism, leading to high energy expenditure. The relationship between diabetes mellitus and ALS may be due to alterations in progranulin levels. An increase in this protein has been observed in ALS patients, whereas in patients with diabetes, this protein is decreased. This alteration in progranulin could explain the neuroprotective effect of diabetes mellitus on ALS [22].

Other risk factors: Other risk factors include exposure to radiation and electromagnetic fields [23], diet, physical activity [24], level of education, trauma, heart factor, age, body mass index (BMI), and metabolic alterations (higher prognosis when it is higher) [25]. Military service is also a risk factor, as studies have shown that soldiers deployed to fight in the Gulf War and exposed to chemical agents are more likely to suffer from ALS than those not deployed [26].

The symptoms of ALS vary, usually manifesting between early ages and more than 50 years. Patients begin to lose muscle strength and coordination. Over time, the abilities to perform daily activities, such as walking, eating, washing, and dressing, weaken to the extent that patients need the help of caregivers or relatives. This decay can start unilaterally in the upper and lower limbs or in activities related to food intake. Initially, symptoms might focus on a hand, arm, or leg but progressively worsen. Patients begin to have breathing problems (risk of choking due to inability to clear mucus), weakness in neck muscles (supporting the head), and communication issues (slower speech and weakened voice). Muscle weakness also leads to stiffness and contraction, swallowing problems, weight loss, and immobility, often resulting in pressure ulcers. Less commonly, patients may experience cognitive impairment, including memory loss and emotional lability. Despite physical limitations, cognitive functions like thinking and reasoning generally remain intact. Because they cannot remove fluids naturally, these patients develop urinary tract infections (due to bladder catheterization). Finally, the patient’s death is usually due to the loss of respiratory function [27].

Due to the lack of effective pharmacological treatments and the complex pathophysiology of ALS, this study aims to provide a comprehensive overview of both pharmacological and non-pharmacological interventions used in ALS management in recent years. Our research seeks to describe current treatment approaches and their outcomes, offering a critical analysis of their efficacy and limitations. By synthesizing this information, we hope to highlight areas of promise and identify gaps in current therapeutic strategies. Ultimately, this study may inspire other researchers to pursue innovative avenues in drug development and explore novel treatment modalities for ALS, potentially leading to improved patient outcomes and quality of life for those affected by this challenging neurodegenerative disorder.

## 2. Materials and Methods

### 2.1. Design and Search Strategy

The review was conducted according to the reporting items for systematic reviews of the PRISMA guidelines [28] and the protocol of the systematic review was registered with PROSPERO under registration number CRD42024569016. An exhaustive bibliographic search was performed using several electronic databases: Dialnet, Scopus (Elsevier), Medline (OVID), and Embase. The search was performed in July 2023 and only articles from the last five years were included. The analyzed equations were “amyotrophic lateral sclerosis AND prevalence or incidence”, “pharmacology AND amyotrophic lateral sclerosis”, “riluzole AND amyotrophic lateral sclerosis”, and “edaravone AND amyotrophic lateral sclerosis” or the keywords “ALS AND excitotoxicity” or “motoneuron disease AND ALS”, “physiopathology AND ALS”, or “neurodegenerative disease AND ALS”. We also performed a search using the Science Citation Index and Scopus to identify reports with citations of the identified articles, and a backward search was performed to revise and retrieve the references of selected studies for the systematic review.

### 2.2. Eligibility Criteria

Studies were included according to the following criteria: (1) clinical studies, clinical trials, and randomized controlled trials; (2) a sample comprising patients with ALS diagnosed by electromyography, muscle mass level, and specific symptomatology (dyspnoea, dysarthria, loss of muscle strength, and loss of mobility) and a healthy population; (3) use of pharmacological treatments during disease; (4) assessment of the improvement in the patient’s quality of life before and during treatment; and (5) full-text access. There were no restrictions on publication date or language.

The population of interest in this review was adult patients of any age and gender diagnosed with Amyotrophic Lateral Sclerosis (ALS). No restrictions by age, gender, ethnicity, geographic location, or other demographic characteristics were imposed on study eligibility. The population of interest comprised patients with sporadic as well as familial ALS, and included the different clinical variants of disease presentation (Primary Lateral Sclerosis and classic ALS). Studies in patients at all stages of ALS progression were included.

The exclusion criteria were as follows: (1) studies including other neurodegenerative pathologies or mixed data, (2) case reports, and (3) no inclusion of pharmacological treatments. We did not place any restrictions on age, sex, or comorbidities to increase the number of results. The type of pharmacological treatment (analgesics or anti-inflammatory drugs) in patients with ALS at the time of diagnosis was not taken into account and was not one of the exclusion criteria. The sporadic or familial origin of the disease was not defined in any of the cases; therefore, it was not considered.

### 2.3. Selection Process: Study Variables and Data Extraction

**Figure 2:**
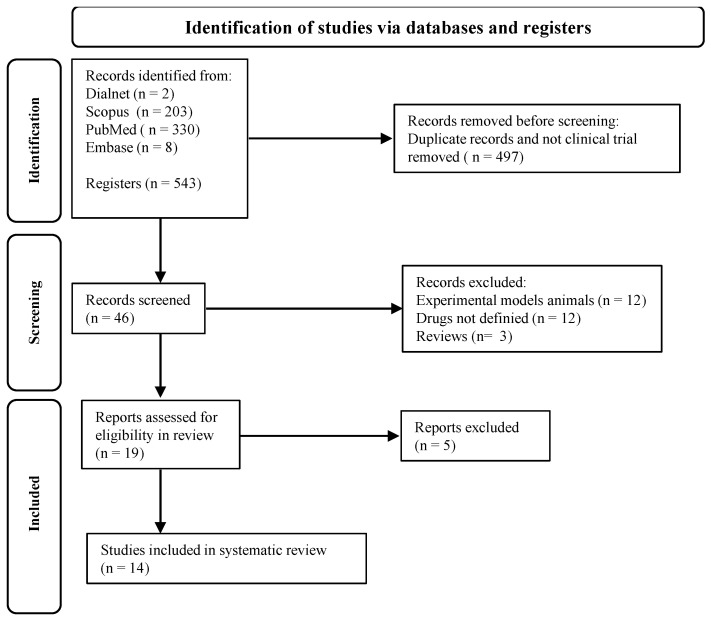
PRISMA flowchart.

Two independent reviewers analyzed the titles, abstracts, and full texts according to the inclusion criteria shown in Figure 2. A third author was consulted in case of discrepancies. All data were extracted and entered into a table by two researchers independently; a third researcher was consulted when there was disagreement. All discrepancies that emerged were resolved by discussion with the rest of the authors, with reference to the original study.

Both pharmacological and non-pharmacological interventions for the treatment of Amyotrophic Lateral Sclerosis were analyzed. Pharmacological interventions of interest included riluzole, edaravone, sodium phenylbutyrate, masitinib, and medications for symptomatic management such as antidepressants, sedatives, muscle relaxants, and medication for hypersalivation, among others. Non-pharmacological interventions evaluated were physical and rehabilitation therapies, occupational therapy, speech and language therapy, nutritional support, respiratory support and non-invasive mechanical ventilation, and multidisciplinary palliative care.

The included studies employed a variety of different comparators or controls, such as placebo, no treatment/standard care, or comparison between different treatment modalities. Given the heterogeneity of interventions and comparators, we did not prespecify a comparator or control of interest for this review. We included studies that evaluated any intervention in comparison to any other control or comparator group.

The following data were extracted from each study: (1) author, year, and location; (2) sample and mean age; (3) purpose of the study; (4) study design; and (5) study results. Corresponding authors were contacted for additional information when necessary. Assessment of the methodological quality and risk of bias of individual studies was performed using the Cochrane Collaboration tool Rob V.2 for randomized controlled trials and the ROBINS-I tool for non-randomized studies [29]. Two reviewers independently conducted data extraction and risk of bias assessment; any discrepancies were resolved by consensus or with the involvement of a third reviewer. The following domains were assessed in each study: randomization methods, allocation concealment, participant/staff masking, outcome assessment masking, incomplete outcome data, selective reporting, and other sources of bias. Assessment was conducted for each main outcome in the studies. Studies were categorized as low risk, high risk, or unclear risk of bias (Table 1) and the analysis was performed with RevMan software 5.3. 

## 3. Results

### 3.1. Pharmacological Treatments

Although there is no cure for ALS, extensive experimentation and numerous clinical trials in recent decades have demonstrated the potential efficacy and long-term utility of various drugs in humans, initially tested in experimental animal models. Riluzole, also known as rilutek, and edaravone, or radicava, are the two most effective drugs studied and approved by the Food and Drug Administration (FDA). Both drugs effectively suppress the progression of clinical manifestations and improve and preserve the quality of life of ALS patients [30,44].

Riluzole, a pharmaceutical agent approved in 1995 by the United States Food and Drug Administration (FDA) and in 1996 by the European Medicines Agency (EMA), was developed by Sanofi-Aventis and was the first drug used to slow the progress of ALS, whose main action is the inhibition of glutamate [45]. Specifically, it establishes a neuroprotective barrier that prevents the excessive stimulation of motor neurons caused by abnormal glutamate production [29]. Although the mechanisms of action of riluzole are not known, with the help of various studies it has been possible to reach the following conclusions: it favors the reabsorption of glutamate by astrocytes and the inhibition of glutamatergic receptors of MNs. Neuronal death is directly proportional to these effects; therefore, if they decrease, neuronal death also occurs. Therefore, if both effects increase, greater neuronal loss occurs. In addition, cell death occurs because the calcium available inside the cell increases considerably, and the mitochondria undergo changes in their composition because the RNA is damaged and the lipids from the membranes of the cell are oxidized [45,46]. The administration of this drug is through oral tablets and by suspension or sublingual oral film (for patients who have severe dysphagia or enteral nutrition) [46]. The dose of oral tablets in patients without dysphagia is 50 mg/12 h (recommended dose) so their intake is approximately two hours post-meal or 30 min pre-meal. This is because when a drug comes in contact with food in the digestive system it decreases the scope of its therapeutic target. Consequently, to maximize the drug’s efficacy, it is recommended to administer it on an empty stomach, avoiding concurrent food intake. Riluzole is the most effective and capable of positively altering the disease, increasing the life expectancy of these patients from up to 3 to 6 months [46]. Side effects include nausea, hepatotoxicity, asthenia, gastrointestinal problems, and an increase in liver enzymes [44].

Edaravone, approved in 2017 by the FDA, was developed by Mitsubishi Tanabe Pharma in Jersey City, NJ, USA. In 2015, it was approved for use in South Korea and Japan. In 2021, it was approved in Thailand, Malaysia, the USA, and China [45]. Although its mechanism of action is not known with clear certainty, several studies suggest that among its functions is to eliminate ROS (antioxidant) and to slow down neuronal and glial oxidative stress, which results in a decrease in the degeneration caused in these patients [45]. The administration of this drug is intravenous (IV) with a dose of 60 mg (intravenous 1 h) using two continuous infusions of 30 mg/100 mL at a rate of 1 mg/min [47]. Also, in several clinical trials, the efficacy of this drug in oral suspension has been analyzed, giving a positive result that should continue to be investigated [35]. It was administered daily in cycles of 14 days with treatment and 14 days without treatment at the beginning. The treatment then moved on to 10-day cycles followed by 14 days off [32]. Notably, the development of an oral formulation of this drug is anticipated to make significant advancements in the near future, potentially mitigating the challenges associated with its current intravenous administration [48]. With regard to the side effects that have been demonstrated, the one that has been most verified is the increase in blood glucose, so the kidneys suffer damage and their absorption capacity is altered, causing its accumulation in urine [31]. We found another type of pharmacology to treat this disease, such as sodium phenylbutyrate and taurursodiol [45]. Both have the ability to reduce the level of stress caused by the endoplasmic reticulum in addition to mitochondrial dysfunction. Importantly, they reduce the death of neuronal characteristics in these patients [33]. The recommended administration method for these drugs is as follows: one sachet in powder form, taken once daily for three weeks, followed by gradual increases in the daily dosage [34]. In addition, the union of dextromethorphan hydrobromide and quinidine sulfate is known as Nuedecta. Dextromethorphan is an antitussive that functions as a glutamate antagonist and neuroprotector. However, quinidine is responsible for inhibiting hepatic cytochrome P450 (enzyme), increasing its half-life in dextromethorphan plasma. Multiple clinical trials demonstrated improvements in patients’ motor functions, including speech, swallowing, and salivary control. Based on these findings, researchers determined that the most effective dosage regimen was 10–20 mg administered twice daily [45].

Mexiletine inhibits excess sodium production, reducing persistent muscle cramps in ALS patients. Nutritional supplements like vitamin E, a potent antioxidant, and creatine, which enhance muscle capacity and potentially slow disease progression, have also been investigated. The combined use of these supplements with targeted pharmacological interventions has been the subject of ongoing research.

### 3.2. Non-Pharmacological Treatments

Given the complex symptomatology of ALS, it is essential to explore non-pharmacological interventions to maximize patients’ functional capacity. Respiratory failure is the most common cause of death in ALS patients. Several randomized trials have concluded that aerobic physical exercise and resistance training with isometric exercises lead to improvements in muscle strength and may reduce injuries by preserving motor skills [35]. These interventions have been shown to decrease muscle stress, reduce fatigue, and potentially slow neuronal degeneration [36]. To maintain adequate lung capacity, assistive devices such as the Lic Trainer (LT), approved in Japan in 2016, have been developed. A study involving 20 ALS patients demonstrated the efficacy of this device in assisting inspiration for patients with severe respiratory system failure [37]. Non-invasive mechanical ventilation (NIV) remains the most widely used therapeutic modality, as it improves quality of life, increases survival, and preserves forced vital capacity in ALS patients, as corroborated by clinical trials [38].

Nutritional interventions have also shown promise. A randomized trial demonstrated the benefits of high-calorie nutritional intake in ALS patients. Muscle weakness, lack of energy, and weight loss are often associated with insufficient calorie intake, particularly in patients with severe dysphagia who require nasogastric tube feeding or parenteral nutrition [39]. While numerous studies have suggested metabolic alterations, specifically dyslipidemia, in ALS patients, conclusive clinical evidence remains limited [25].

As both respiratory and motor functions are affected in ALS, speech and language impairments are common. A study with four patients visualizes the need and satisfaction of adaptation techniques to be able to communicate in some way, through boards, drawings, etc. Similarly, the ability to maintain even minimal oral food intake was found to be more satisfying for patients than exclusive tube feeding [40].

**Figure 3:**
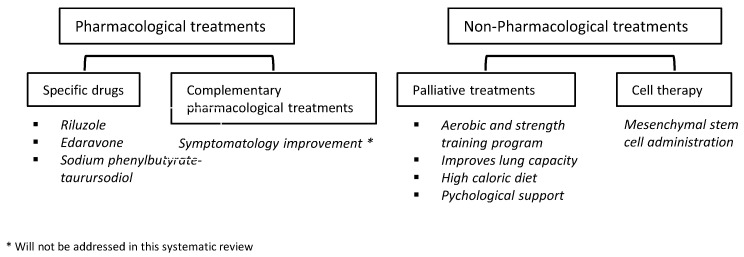
Treatments for ALS.

Another therapy involves the use of mesenchymal stem cells to help slow the progression of the disease. In one of the trials that we observed, after experimentation with 15 patients, of whom only 8 were analyzed, if the patients carried a rapid progression of the disease, the treatment with this type of cells benefited them, unlike those with slow disease progression since no important variations were observed [41]. To corroborate that stem cells are effective for ALS patients, more clinical trials with patients are needed, as they have been scarce so far. It should be noted that in another trial where patients were treated with autologous MSCs, no major adverse events developed, and, therefore, they remain safe practices for patients [42]. A summary diagram of the different treatments for ALS is described in Figure 3.

Finally, a crucial aspect that warrants attention is the role of caregivers for ALS patients, whose function is vital for the survival of those affected. Caregivers, ranging from partners to health workers and family members, are essential contributors to patient care, especially when individuals can no longer perform basic tasks independently. One study examined caregivers’ responses to a psychosocial support workshop, which included psychological exercises, psychoeducation, and practical life tips under the supervision of a psychologist [43]. Many caregivers experienced distress, highlighting the emotional and psychological challenges they faced. These studies provide insights into the emotions, thoughts, and uncontrollable situations that caregivers encounter in their daily lives. Therefore, providing comprehensive information and support is crucial to fostering a healthy patient–caregiver relationship that benefits both parties.

Table 2 presents all the data and results. The first section of the table describes five studies on pharmacological treatments, followed by seven studies on non-pharmacological interventions.

## 4. Discussion

Although great advances are being made in studies aimed at patients with ALS, the existing enigmas about this disease remain high, causing many situations in which the approach is complicated. The priority of these patients is to receive efficient support and increase their quality of life. This supports the idea of generating a correct multidisciplinary approach, where each of the interventions carried out is the result of a better future. As we have already seen, the support mentioned above can range from a variety of drugs to treat both the symptoms and progression of the disease, psychological support, family support, nutritional care, muscle cramping control, and respiratory care. However, because the pathophysiology of the disease is not precisely known, it is difficult to design efficient drugs that stop both neuronal degeneration and extend the half-life of patients.

Riluzole is one of the star drugs in most of the clinical trials analyzed. Furthermore, adjunctive therapies to riluzole have been identified; however, their efficacy remains subject to debate. One pharmacological agent that has been investigated in combination with riluzole is masitinib, an oral tyrosine kinase inhibitor, which was first investigated in rat models and human patients with ALS, increasing their life span and slowing the progression of the disease. It was found to play a role in neuroprotection, such as immunomodulatory activity [49,50].

Although mesenchymal stem cells are still being studied, a type of CD133 stem cell has been found whose function equals or exceeds that of the previous ones [51]. The first was performed in the spinal cord and later carried out in the primary motor cortex. H Deda and S Kocabay used stem cells in their study but these were hematopoietic progenitors of bone marrow and the location to be inserted was not limited to the spinal cord but also to the end of the brainstem, resulting in improved quality despite the fact that in the trial not all participants survived [52]. Gene therapy with the aim of decreasing the expression of the genes responsible for the disease has also been used. Tofersen is an antisense RNA designed to reduce the production of the mutant SOD1 protein, showing promising results in clinical trials [53]. Other gene therapies are based on editing mutant genes, as is the case with SOD1, or the use of vestibules for the introduction of genes encoding neurotrophic factors [54].

The progression of Amyotrophic Lateral Sclerosis (ALS) is characterized by persistent pain, prompting research into pharmacological interventions such as mexiletine for cramp management and muscle function enhancement. However, direct pain management remains underexplored in clinical trials. One notable study investigated the efficacy of intrathecal baclofen pumps in alleviating pain associated with immobility, rigidity, and spasticity, with eight participants reporting complete pain resolution [55].

Respiratory failure constitutes a primary mortality factor in ALS patients. Research has demonstrated that non-invasive ventilation (NIV) can prolong survival and enhance the quality of life in ALS patients, although these benefits were not observed in individuals with severe bulbar impairment [56]. While diaphragmatic stimulation systems can be utilized in conjunction with NIV, several researchers posit that the simultaneous application of both interventions may decrease patient longevity [57]. Furthermore, a clinical trial suggested that diaphragmatic training techniques yield no positive outcomes and may even precipitate a decline in respiratory function among those who mastered the technique, with few patients successfully modifying their respiratory patterns [58]. The study conducted by González Bermejo and Similowski corroborated these findings, advising against diaphragmatic stimulation and deeming it contraindicated during the initial respiratory phase [59].

Nutritional management in ALS diverges from recommendations for the general population. While calorie restriction is typically advised to mitigate obesity risk, ALS patients benefit from a high-fat diet [60]. Malnutrition is prevalent among ALS patients, necessitating adequate caloric intake. Dorst and Ludolph’s randomized controlled study involving 64 patients demonstrated that a diet supplemented with high-calorie or ultra-high-calorie fat was optimal for weight gain, despite causing gastrointestinal discomfort. Conversely, carbohydrate-rich supplements led to appetite suppression, although weight gain was still observed, albeit to a lesser extent [61].

The limited applicability of animal model research to human ALS cases stems from the fact that only 10% of cases are familial. Experimental animal models for ALS are predicated on mutations or genetic modifications of genes implicated in the disease. Over 30 genes associated with familial ALS subtypes have been identified, with sod1g93a, tdp-4, and c9orf72 being particularly prominent [62]. Numerous studies utilizing these animal models have been conducted across various laboratories, focusing on the primary theories underlying ALS pathophysiology. The majority of these investigations have sought to elucidate the molecular mechanisms responsible for excitotoxicity-induced neuronal death, either by reducing the intrinsic excitability of motor neurons or by diminishing their sensitivity to glutamate. This approach aims to decipher the various signaling pathways that render motor neurons more susceptible to degeneration. Despite promising results in animal models, the translation of these therapeutic targets to human ALS patients remains a significant challenge, necessitating further research and development.

## 5. Conclusions

The need for developing novel therapies and strategies to treat ALS more effectively and efficiently is evident. Among the drugs discussed in this systematic review, only riluzole and edaravone have demonstrated significant positive outcomes, delaying disease progression by several months. A complementary therapeutic approach involves focusing on palliative measures to support the neurodegenerative process, aiming to maximize patient comfort. It is crucial to emphasize the importance of alleviating symptoms and acknowledging the caregiver’s role, highlighting the vital importance of symptom management medications. Further clinical studies investigating emerging drugs are necessary, as well as research into potential combination therapies targeting different aspects of ALS pathophysiology. These efforts may lead to more comprehensive and effective treatment strategies for ALS patients.

## Figures and Tables

**Table 1:** Risk of bias.

Study	Random Sequence Generation	Allocation Concealment	Blinding of Participants and Personnel	Blinding of Outcome Assessment	Incomplete Outcome Data	Selective Reporting	Other Bias
Brooks et al., 2019 [30]	Low	Low	Low	Low	Low	Low	Low
Witzel et al., 2022 [31]	Low	Low	Low	Low	Low	Low	Low
Shimizu et al., 2021 [32]	Low	Low	Low	Low	Low	Low	Low
Paganoni et al., 2021 [33]	Low	Unclear	Low	Low	Low	Low	Low
Paganoni et al., 2020 [34]	Low	Low	Low	Low	Low	Low	Low
Kalron et al., 2021 [35]	Low	Low	Low	Low	Low	Low	Low
Meng et al., 2020 [36]	Low	Low	Low	Low	Low	Low	Low
Yorimoto et al., 2020 [37]	Low	Low	Low	Low	Low	Low	Low
Rudnicki et al., 2021 [38]	Low	Low	Low	Low	Low	Low	Low
Ludolph et al., 2020 [39]	Low	Low	Low	Low	Low	Low	Low
Luchesi et al., 2018 [40]	Low	Unclear	Low	Low	Low	Low	Low
Siwek et al., 2020 [41]	Low	Low	Low	Low	Low	Low	Low
Petrou et al., 2021 [42]	Low	Low	Low	Low	Low	Low	Low
De Wit et al., 2019 [43]	Low	Low	Low	Low	Low	Low	Low

**Table 2:** Studies used to carry out the systematic review of pharmacological and non-pharmacological treatments.

Title	Author/S, Year and Country	Objectives	Studio Design	Results
Riluzole oral suspension: Bioavailability following percutaneous gastrostomy tube-modeled administration versus direct oral administration	Benjamin Rix Brooks, Paolo Bettica, Sara Cazzaniga, October 2019, Italy [30]	“To compare the bioavailability of riluzole oral suspension by intragastric tube with healthy fasting patients”	Clinical study, randomized controlled trial Patients with ALS and healthy volunteers (62 patients in total) were included	It was concluded that intragastric tube and administration of riluzole have positive effects and can avoid percutaneous endoscopic gastrostomy tubes.
Safety and effectiveness of long-term intravenous administration of Edaravone for treatment of patients with Amyotrophic Lateral Sclerosis	Simon Witzel, André Maier, Robert Steinbach, Thomas Meyer, Albert C Ludolph, February 2022, Germany [31]	“To assess the long-term safety and effectiveness of intravenous edaranova therapy for ALS patients in a real-world clinical setting”	Clinical study, randomized controlled trial 324 selected patients where 194 patients were treated intravenously and 130 patients with ALS received standardised therapy	“We concluded that intravenous edaravone was well tolerated and reliable without any disease modification”. Compared with standard treatment, intravenous treatment does not have any extra support value. No differences were observed in terms of survival, ventilation, or disease progression.
Evaluation of pharmacokinetics, safety and drug interactions of an oral suspension of edaravone in healthy adults	Hidetoshi Shimizu, Yukiko Nishimura, Yoichi Shiide, Kazuoki Kondo, March 2021, Japan [32]	Study 1: “To evaluate the pharmacokinetics, safety and tolerability of single doses of oral edaravone in healthy adult males” Study 2: “Evaluating drug interactions, safety and tolerability of various drugs with edaravone in healthy adult men” Evaluate the same aspects with multiple doses of edaravone alone	Clinical study, 2 phase 1 studies with healthy Japanese patients aged 20–45 years	Positive results were observed in both cases, without manifesting problems when administered alone or with other medications. Endoplasmic exposure in both studies showed no significant differences.
Long-term survival of participants in the CENTAUR trial of sodium phenylbutyrate–taurursodiol in amyotrophic lateral sclerosis	Sabrina Paganonni, Suzanne Hnedrix, Samuel P Dickson, Newman Knowlton, Merit E Cudkowicz, October 2020, Massachusetts [33]	To analyze patient survival in CENTAUR over a long period of time Comparison of PB-TURSO with CENTAUR	Clinical study, randomized controlled trial with 137 patients	Confirmation, along with an earlier study (randomized controlled trial) that PB-TURSO has survival-related benefits in patients with ALS. Compared with CENTAUR with PB-TURSO, the latter has greater benefits. Patients on CENTAUR had a lower death rate than those on a randomized placebo.
Trial of sodium phenylbutyrate–taurursodiol for amyotrophic lateral sclerosis	Paganoni Sa, Macklin A T, Berry JD, Cudkowicz ME, September 2020, Massachusetts [34]	Describe the efficacy and safety of the compounds in Amyotrophic Lateral Sclerosis	Clinical trial, 177 ALS patients with 137 randomized to receive sodium phenylbutyrate–taurursodiol (89 members)/placebo (48 members)	The placebo produced a faster deterioration at the functional level than the compound analyzed.
Effects of a 12-week combined aerobic and strength training program in ambulatory patients with amyotrophic lateral sclerosis: a randomized controlled trial	Kalron Al, Mahameed Ib, Weiss Israel, Kramer Reuven Mordechai, January 2021, Israel [35]	“To compare the effectiveness of a combined program of aerobic, strength, and flexibility training with flexibility alone in patients with ambulatory ALS”	Clinical study, randomized controlled trial, 32 outpatients with ALS were included in the first group of aerobic and other stretching exercises	Flexibility alone has a much smaller impact on patients with ALS than aerobic strength exercise. Strength training, along with aerobics, has a greater impact on improving breathing, movement, and well-being.
Effects of exercise in patients with amyotrophic lateral sclerosis	Lijiao Mneg, Xiaoxiao Li, Qiang Gao, September 2020, China [36]	Systematic review of the efficacy and safety of exercise in ALS patients	Meta-analysis/systematic review, 7 randomized controlled trials involving 322 ALS patients	Both the functional capacity and lung capacity of ALS patients were improved with the help of physical exercise. Aerobic exercise resulted in more favorable outcomes.
Lung insufflation capacity with a new device in amyotrophic lateral sclerosis: Measurement of the lung volume recruitment in respiratory therapy	Yorimoto Keisuke, Ariake Yo, Kobayashi Yoko, May 2020, Japan [37]	“To validate the usefulness of measuring pulmonary insufflation capacity with the LIC TRAINER in ALS patients”	Clinical study, retrospective study, 20 patients undergoing respiratory therapy from 2014 to 2017	LIC TRAINER is a very helpful device as it would be easier to manage respiratory therapy in these patients.
Noninvasive ventilation use by patients enrolled in VITALYTY-ALS	Rudnicki Stacy A, Andrews JA, Bian A, Cockroft SM, Shefner JM, April 2021 [38]	“To assess non-invasive ventilation (NIV) prescribing practices and patient compliance during VITALITY-ALS”	Clinical study, randomized controlled trial, 565 patients on placebo or tirasemtiv; a total of 195 patients received non-invasive ventilation	These patients, 179/565 had 50% less vital capacity compared to the others, in addition to a decrease in respiratory and sleep symptoms. Of the 179 patients, 122 were prescribed non-invasive ventilation.
Effect of high-caloric nutrition on survival in Amyotrophic Lateral Sclerosis	Ludolph CF, Dorst JJ, Dreyhaupt J, Weishaupt HH, Kassubek JJ, Dupuis T, February 2020, Germany [39]	“To evaluate the efficacy of a high-calorie fat diet in increasing survival”	Clinical study, randomized controlled trial, A comparative study involving 301 patients was conducted to analyze gender-specific differences between male and female from February 2015 to September 2018.	The placebo group had a survival value of 0.39. The group fed a high-calorie fat diet had a survival value of 0.37. Both groups received treatment for 28 months. There was no strong evidence of benefits of this treatment in patients in whom the disease increased rapidly.
Palliative care, Amyotrophic Lateral Sclerosis and swallowing	Luchesi Fontes K, Silveira Coast, August 2018, Brazil [40]	“To discuss speech and language pathology therapy intervention in dysphagia with a focus on palliative care and quality of life”	Case report, case study conducted on 4 patients with ALS	Subjects diagnosed with mild dysphagia reported increased comfort levels, exhibited positive affective responses, and demonstrated improved capacity for oral mastication and deglutition of minimal quantities of food. The use of nutritional mechanisms, such as tubes, causes discomfort. In cases of severe dysphagia, this would not be possible.
Repeat Administration of bone marrow-derived mesenchymal stem cells for treatment of amyotrophic lateral sclerosis	Siwek T, Jezierska-Wozniak K, Badowska W, Maksymowicz Wojciech, 2020, Poland [41]	“Investigating repeated intrathecal injection of autologous bone marrow-derived mesenchymal stem cells into patients for the treatment of ALS”	Clinical study, 15 patients between 18 and 69 years of age with sporadic ALS; use of the ALSFRS-R (Revised Amyotrophic Lateral Sclerosis Functional Rating Scale)	The safety profile of the intervention was substantiated by the observation that adverse events were limited to a single patient, who experienced a transient headache that resolved spontaneously and without sequelae. Efficacy was poor due to no or near-significant efficacy because the rates of progression did not decrease.
A phase II clinical trial with repeated intrathecal injections of autologous mesenchymal stem cells in patients with amyotrophic lateral sclerosis	Petrou Panayiota, Kassis Ibrahim, Yaghmour Nour, Ginzberg A, Karussis Dimitrios, 2021, Jerusalem, Israel [42]	“To evaluate the safety and efficacy of repeated intrathecal administrations of autologous MSCs in ALS patients”	Phase II clinical trial, 20 subjects aged between 20 and 70 years with ALS	It has been corroborated that the injections used in patients are safe and have clinical benefits. Adverse events did not occur.
User perspectives on a psychosocial blended support program for partners of patients with Amyotrophic lateral sclerosis and progressive muscular atrophy	Jessica de Wit, Sigrid C.J.M Vervoort, Eefke van Eerden, Leonard H. van den Berg, Johanna M.A.Visser-Meily, Anita Beelen, Carin D.SchrÖder, 2019, Netherlands [43]	“Collect information on experiences with different components of the support program” Analyze information collected by caregivers when following the program	Clinical study, randomized controlled trial Interviews of 23 caregivers of patients with ALS	The intervention program for caregivers received a favorable assessment from participants. Prior to program implementation, valuable data regarding caregivers’ emotional states and cognitive processes were successfully collected. As a result of the intervention, a significant improvement was observed in caregivers’ ability to effectively manage patient care.

## Data Availability

The data presented in this study are available on request from the corresponding author (matoga@ugr.es).
